# Understanding Depression Symptom Heterogeneity in South Asian Minority Groups: A Systematic Scoping Review

**DOI:** 10.1192/bjp.2026.10539

**Published:** 2026-03-10

**Authors:** Rose Rickford, Mel Ramasawmy, Rachel Francois-Walcott, Andrea Martinez, Madiha Sajid, Odelia Obasoyen, Areeba Shahab, Firoza Davies, Hannah Frith, Paramjit Gill, Hameed Khan, Amy Ronaldson, Tanveer Siyan, Lydia Poole

**Affiliations:** 1 School of Psychology, University of Surrey, Guildford, GU2 7XH, UK; 2 Wolfson Institute of Population Health, Queen Mary University of London, Charterhouse Square, London, EC1M 6BQ, UK; 3 PAPER Study Public and Patient Involvement Representative; 4 Institute of Psychiatry, Psychology, and Neuroscience, King’s College London, London, SE5 8AB, UK; 5 Warwick Applied Health, University of Warwick, Coventry, CV4 7AL, UK

## Abstract

**Background:**

Depression is the most common mental illness globally and is a leading cause of years lived with disability. The manifestation of depressive symptoms can vary between ethnic groups. Individuals in South Asian countries experience higher levels of somatic symptoms than those in other regions. It is not known whether this pattern extends to the South Asian diaspora.

**Aims:**

To provide a qualitative synthesis of what is known regarding depression symptoms among the South Asian diaspora in English-speaking countries.

**Methods:**

A systematic scoping review was conducted following PRISMA-Scr guidelines based on a pre-registered protocol (doi.org/10.17605/OSF.IO/5E6ZK). The review included qualitative, quantitative, and mixed-methods primary research reporting depression symptoms based on samples of adults of the South Asian diaspora in English-speaking countries with substantial South Asian populations. Qualitative content analysis was used to identify widely reported symptoms of depression among the South Asian diaspora.

**Results:**

Commonly reported symptoms included physical pain, heart-related symptoms, and repetitive negative thinking, none of which are included in ICD-11 diagnostic criteria for depressive disorders. Sleep-related disturbances are also widely reported in research into experiences of depression among the South Asian diaspora.

**Conclusion:**

Current diagnostic criteria for depression may not capture symptoms of some South Asian patients, which may cause missed opportunities for intervention.

## Introduction

Depression is the most common mental illness globally, affecting over 300 million people world-wide.^1,2^ It is a leading cause of years lived with disability.^2^ The manifestation of depressive symptoms can vary across ethnic groups, often influenced by cultural notions of distress, religious beliefs, social norms, and language.^3^ A systematic review of depression symptoms worldwide found that individuals in South Asia had a higher prevalence of somatic symptoms – bodily sensations and physical dysfunctions^4^ –compared to other regions.^5^ In South Asian countries, four of the five most common depression symptoms reported were somatic, including “headaches and issues with the heart”, which are not recognised in International Classification of Diseases (ICD)-11 diagnostic criteria.^5–7^ Only one of the two cardinal symptoms listed in ICD-11 – low mood – was ranked in the top five. There is a gap in knowledge of symptom experiences in South Asian diaspora populations, which might be influenced by adaptation to a new cultural environment (acculturation). There is also a lack of understanding of differences in depression symptomology between different South Asian populations, including different ethnicities represented in the South Asian diaspora.^8^ Awareness of different clinical presentations is necessary for accurate diagnosis, so understanding symptom heterogeneity is important for combatting health inequalities.

In the United Kingdom (UK), South Asian groups^i^ form the largest minority ethnic population.^9^ A systematic review found higher rates of total depressive symptoms for South Asian compared with White British groups.^10^ However, despite consulting with their General Practitioner (GP) more frequently than their White counterparts, Pakistani women in the UK have been found to be less likely to receive treatment for depression.^11^ Ethnic differences in depressive symptomology, and associated problems with the validity and reliability of diagnostic screening tools, may be a factor in explaining such findings.

This review aims to explore reported depression symptoms among the South Asian diaspora, comparing these to ICD-11 diagnostic criteria, considering acculturation and ethnic differences. It forms part of the NIHR funded ‘Prescribing Antidepressants in Primary care: Ethnic inequalities in tReatment’(PAPER) Study, which is investigating symptoms and treatment of depression among people of South Asian populations in the UK.^12^ Evidence suggests that there may be a relationship between language and reported depression symptoms,^13^ so in order to maximise relevance to the UK-context, this review focuses on symptomology among South Asian populations in English-speaking countries with large South Asian diasporas.

## Methods

A systematic scoping review was selected as the appropriate method because it allows a systematic search strategy in order to produce evidence synthesis for broad research questions, with the inclusion of a wide range of different types of literature.^14^

### Search strategy and selection criteria

The review was conducted following the Preferred Reporting Items for Systematic Reviews and Meta-Analyses for Scoping Reviews (PRISMA-Scr) guidance.^15^ It included original qualitative, quantitative, and/or mixed method studies, written in English, that were based on findings from adult participants of the South Asian diaspora living in the UK, USA (United States of America), Canada, Australia and/or New Zealand,^ii^ where reported findings included details of symptoms of study participants experiencing depression (diagnosed or self-identified), or mental distress where authors identified symptoms as being potential indicators of depression. Studies were excluded if South Asian-specific findings could not be extracted from wider demographic categories, and, for quantitative studies, where depression symptoms were not reported item-by-item. Studies in which depression was considered alongside another disease, such as diabetes or cancer, were also excluded, as were studies that focused solely on postpartum depression. There was no limit on publication date. The review protocol was pre-registered with Open Science Framework (OSF).^16^ A detailed search strategy is presented in Appendix 1.

### Information sources

Database searches of SCOPUS, EMBASE, PubMed, PsychInfo, Web of Science, Google Scholar, ProQuest and The Kings Fund were conducted in March-May 2024 and updated in July-October 2024 (RFW, RR). Search terms were identified to target three core concepts: depression; South Asian; and people living in the UK, USA, Canada, Australia and New Zealand. Search terms designed to target these concepts were piloted for validity before the final searches were conducted.

### Study selection

Search results were deduplicated in Endnote^17^ and then imported into Rayyan (rayyan.ai) ^18^ for screening. Titles and abstracts were screened against inclusion and exclusion criteria (RFW, OO, RR), with 10% of papers reviewed by two reviewers. Full text screening was conducted (RFW, OO, AM, RR), with at least two authors screening each paper. Throughout the screening process, disagreements were resolved first through discussion between two reviewers, followed by consultation with a third reviewer (LP/MR) if consensus could not be reached. Once an initial list of full texts had been identified, forward and backward citation searching was carried out (RFW, RR) to identify further relevant studies.

### Data analysis

A data charting form (see Appendix 2) was drafted by RR in consultation with AM, LP and MR. The form was piloted by RR and AS using five randomly selected studies to check consistency between authors. A summary of charted data is provided in Appendix 3.

Qualitative content analysis,^19^ an established method for use in reviews,^20^ was used to identify: i) reported symptoms and common ways of describing or expressing distress and depression experiences, ii) symptom comparisons between ethnicities. Studies were categorised into three groups: A) those in which an overall list of symptoms/symptom prevalence in the sample was presented; B) those in which symptoms were analysed in comparison to a White majority population in the country where the study was based; and C) those in which symptoms were only mentioned in passing or in data extracts while presenting other findings.

To identify reported symptoms and ways of describing and expressing distress and depression, findings and quotations from all papers were coded using NVivo.^21^ Coding captured symptoms that are not currently widely recognised as indicative of depression as well as those that are. For qualitative studies, in which participants described their experience in their own words, all reported symptoms were coded.^iii^ For quantitative studies, where participants selected symptoms from a pre-determined list, the most frequently selected symptoms were coded.^iv^ To identify symptoms that are more common among South Asian populations than other ethnic groups, comparative findings from Group B articles were additionally charted in Excel. The final principal findings were developed by comparing codes and charting from the two sub-analyses to identify widely reported depression experiences among South Asian diaspora populations, including when compared to other populations, which are not currently recognised in diagnostic criteria for major depressive disorder.

## Findings

### Papers selected for review

**Figure 1:**
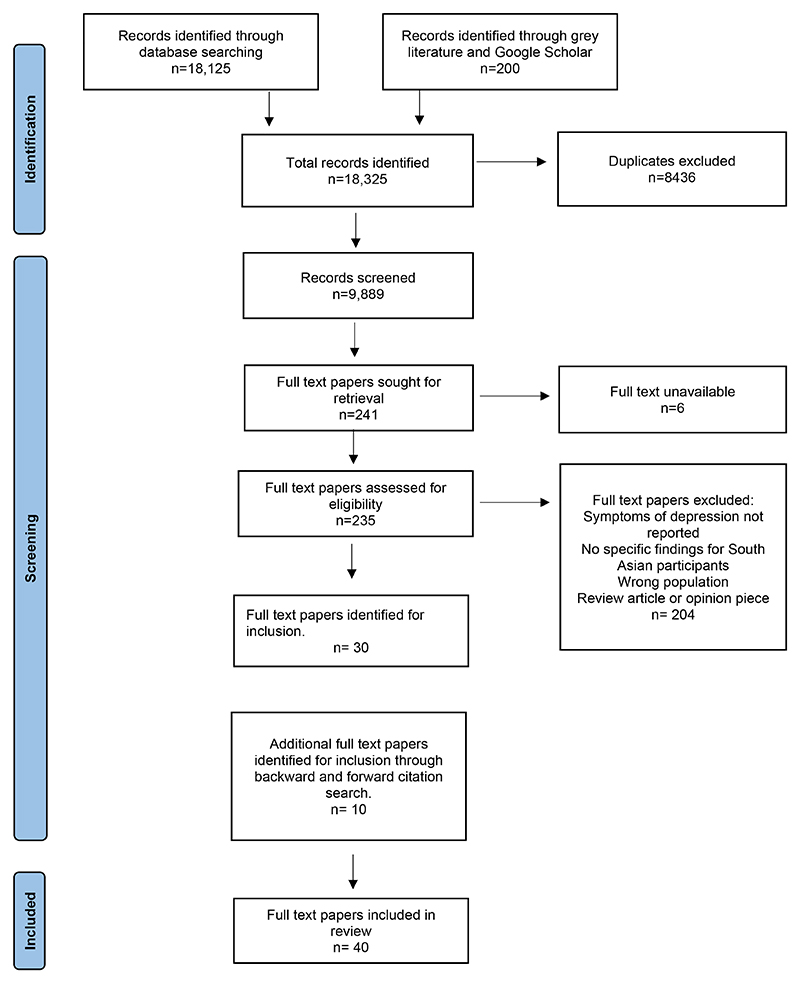
PRISMA flow diagram

A total of 9,689 unique papers were identified through searches. Forty were selected through screening (n=30) and forward and backward citation searching (n=10), of which 26 were qualitative, 13 were quantitative, and one used mixed-methods. See Figure 1 for a PRISMA flowchart of the screening process.

The included papers reported research conducted in the UK (n=25), Canada (n=9), USA (n=4), and Australia (n=2), with participants of Pakistani (n=15), Indian (n=14), Punjabi (n=8), Bangladeshi/Bengali (n=5), Sri Lankan (n=1), Tamil (n=1), Nepalese (n=1), and Afghan (n=1) heritage.^v^ Remaining studies focused on non-specified South Asian diaspora populations (n=8). Of the papers that reported the birth country of participants (n=27), the majority (85%) of participants were first-generation immigrants from South Asian countries.

**Figure 2:**
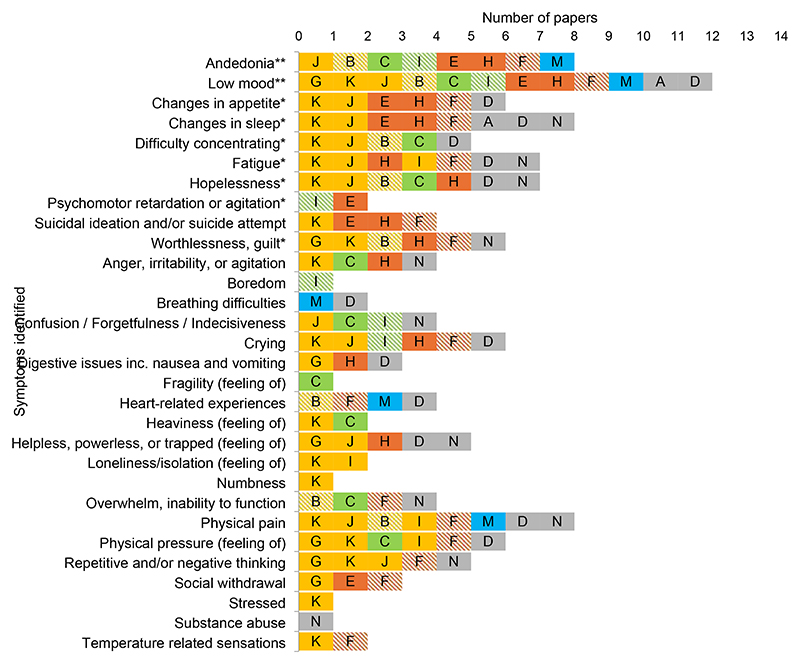
Symptoms of depression among South Asian diaspora samples (Group A papers) Ethnicity 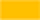
 Pakistani 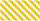
 Multiple South Asian ethnicities with Pakistani majority 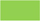
 Indian 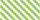
 Multiple South Asian ethnicities with Indian majority 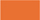
 Punjabi^†^ 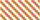
 Multiple South Asian ethnicities with Punjabi majority 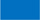
 Bengali^††^ 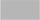
 Unspecified South Asian, or multiple South Asian ethnicities with no overall majority Study details A. Ahmed et al. (2017) (quant***; n=50; sex F:M=100:0; age <45=66%, >45=34%) B. Akram (2012) (qual****; n=13; sex F:M=64:36; age mean=38) C. Brijnath & Antoniades (2018) (qual; n=28; sex F:M=54:46; age range=19-84) D. Burr (2002) (qual; n=46; sex F:M 100:0; age mean=37) E. Chalal (2018) (qual; n=6; sex F:M=83:17; mean=43) F. Fenton & Sadiq‐Sangster (1996) (qual; n=59; sex F:M=100:0; age range = 20s-60s) G. Gask et al. (2011) (qual; n=15; sex F:M=100:0; age mean=42) H. Grewal (2009) (qual; n=6; sex F:M=83:17; age mean=28) I. Lai & Sarood (2008) (quant; n=210; sex F:M=44:56; age mean=66) J. Malik (1997) (quant; n=120; sex F:M=50:50; age range=25-60) K. Mallinson & Popay (2007) (qual; n=31; sex F:M=52:48; age range=19-65) L. Rafique (2010) (qual; n=7; sex F:M=100:0; age mean=36) M. Silveira & Ebrahim (1995) (quant; n=75; sex F:M=31:69; age mean=64) N. Yusuf (2019) (qual; n=15; sex F:M=40:60; age mean=37) * Symptom included in ICD-11 diagnostic criteria ** Cardinal symptom in ICD-11 diagnostic criteria *** Quantitative **** Qualitative ^†^ Punjabi refers to people with cultural heritage from the Punjab region, which spans South East Pakistan and North West India. Punjabi is also a language spoken in this region and among its diaspora. ^††^ Bengali is a language spoken in Bangladesh and in the Eastern Indian states of West Bengal, Tripura, and Assam, and among the respective diasporas.

Twenty-two (22) of the papers (11 qualitative, 11 quantitative) included an authors’ analysis of depression symptoms in South Asian participants.^22–43^ This included 14 studies (ten qualitative, four quantitative) reporting depression symptoms among a South Asian sample (Group A) (Figure 2), and 11 studies (eight quantitative, three qualitative) comparing symptomology of South Asian and White samples (Group B) (Table 1). Three studies were in both groups.^25,32,43^

The remaining 18 papers focused their analyses on other topics (e.g. depression coping strategies; barriers to accessing care; cultural conceptions of mental illness) and included ad-hoc content on depression symptoms while reporting other findings.^44–61^ This content was coded and contributes to the full list of symptoms featured across reviewed literature (Table 2), and is drawn upon in the written analysis.

### Principal findings

Qualitative content analysis generated a list of symptoms reported across the literature (Table 2). Three principal findings are outlined in detail below. These are symptoms or groups of symptoms reported widely across the literature in both qualitative and quantitative studies, including studies comparing South Asian diaspora populations to White populations. The first finding is that somatic symptoms of depression are prevalent, particularly sleep disturbance, which is listed in the ICD-11, and physical pain, which is not. Second, individuals describe their depression using language relating to the heart. Third, there is a prevalence of repetitive negative thinking which may be related to trauma.

Our analysis also identified multiple reports of “tension.” ^24,32,35,43,44,47,60^ Rather than being presented as a symptom of depression, this was described as similar to enduring stress, and is therefore not included as a theme, but is summarised in Appendix 4.

### Principal Finding 1: Somatic symptoms of depression

Figure 2 maps the number of papers identifying different ICD-11 and non-ICD-11 features of depression in 14 papers reporting symptoms among South Asian participants (Group A). This includes 10 qualitative papers examining experiences reported by participants during interviews,^24–29,32,33,35^ and four quantitative papers reporting symptoms measured using questionnaires.^23,31,34,43^ The cardinal symptom of low mood was reported in the most papers (12/14).^23–34^ However, the cardinal symptom of anhedonia was reported in the same number of papers (8/14) ^24,25,27,28,31,33,34,47^ as non-cardinal somatic symptoms of sleep disturbance (8/14) , ^23,26–28,30,32,33,35^ and physical pain (8/14),^24,26,28,32–35,43^ one of which (pain) is not listed in ICD-11. Reports of sleep disturbance included difficulty falling asleep, early morning awakening, and disturbed sleep. Reports of pain included unspecified physical pain, body aches and pains, neck pain, and headaches. Other somatic symptoms reported included nausea and vomiting,^26,29,30^ sensations related to the heart,^24,26,28,34^ temperature related sensations,^28,32^ feelings of heaviness,^25,32^ feelings of numbness,^32^ and sensations of pressure, squeezing, or squashing.^25,26,28,29,32^ Somatic symptoms were also reported in quotations provided in the 18 papers in Group C in which authors did not specifically analyse depressive symptoms (in studies including Pakistani, Indian, Bangladeshi, Punjabi, Sri Lankan, Afghan, Tamil, and unspecified ‘South Asian’ participants), ^44–49,51,55,57–60^ with participants reporting, for example “terrible headache”, ^56^ “felt sick”, ^44^ burning sensations^47^, dizziness and fainting, ^24,49,56^ and hair loss.^45^

Table 1 reports the 11 papers (eight quantitative, three qualitative) which compared symptoms between South Asian and White participants (Group B). Only the two most recent of these reported their findings in relation to ICD or DSM (Diagnostic and Statistical Manual of Mental Disorders)^7^ symptoms, and neither found a significant difference in somatic symptoms between South Asian and White patients. These included a 2017 qualitative study comparing Anglo-Australian and Sri Lankan depression patients living in Australia,^36^ and a 2020 quantitative study using clinical records of South London (UK) depression patients aged over 65 years.^22^ However, earlier studies comparing South Asian and White participants included symptoms not listed in the ICD, and overall these studies indicate a possible pattern of South Asian individuals experiencing higher rates of somatic symptoms, compared to White individuals. A 2007 qualitative study used semi-structured interviews and distress screening tools (Self-Reporting Questionnaire (SRQ);^62^ Schedules for Clinical Assessment in Neuropsychiatry (SCAN);^63^ Life Events and Difficulties Schedule (LEDS)^64^) to explore representations of distress among Pakistani and White people in northwest England. While the authors reported no clear difference in prevalence of somatic symptoms overall, Pakistani participants reported temperature changes, and feelings of pressure and heaviness in the head, which White patients did not.^32^ A 1996 quantitative study compared rates of depression symptoms (measured through the Hospital Anxiety and Depression Scale (HADS))^65^ and somatic symptoms (measured through the Bradford Somatisation Index (BSI))^66^ among White and Asian (predominantly Pakistani and Indian) patients at two GP practices in England. Affective and cognitive elements of depression measured using the HADS were similar between groups, but Asian patients were more likely to report a “weak or sinking heart", “aches and pains all over the body", and "a feeling of heat inside your body" on the BSI.^42^ A 2004 study measured symptoms among White English and Punjabi primary care patients in London using the General Health Questionnaire (GHQ-12)^67^ and the Amritsar Depression Inventory (ADI),^68^ which was developed in the Punjab region of India and contains somatic symptoms not included in the GHQ-12.^39^ The researchers also conducted a second stage interview which included questions about somatic symptoms. They found that Punjabi patients with somatic symptoms (defined as “any sort of ache or pain, for example headache or indigestion, or any other sort of bodily discomfort" that the patient considers is "due to, or made worse by, feeling low, anxious or stressed"), were more likely to report depression than White English patients with somatic symptoms defined in the same way,^39^ although another paper from the same team reported that the ADI item “I feel sad because of aches and pains” was a predictor of depression cases in White English patients and not in Punjabi patients.^37^ (We suggest that presence of physical illnesses may affect responses to this latter item, but physical illnesses were not reported in the paper so their impact cannot be assessed). A 1997 thesis used the GHQ-28 to compare symptoms of Pakistani people in Britain to both a White British sample and a sample of Pakistani people living in Pakistan.^43^ The GHQ-28 includes somatic symptoms such as temperature-related sensations, headaches, and feeling of pressure in the head, and when using this tool, the study found that Pakistani people living in Britain had similar levels of somatic symptoms to those living in Pakistan, which were higher than the White British sample.

Overall, our analysis paints a picture of South Asian diaspora patients experiencing a range of somatic symptoms of depression, with sleep changes and physical pain reported in the most studies. Studies directly comparing recognised symptoms among South Asian and White participants did not find that sleep changes, or any ICD somatic symptoms, were more prevalent among South Asian participants. However, other somatic symptoms not captured in ICD, such as physical pain, sensations of pressure and heaviness, and temperature-related sensations, were found to be more prevalent among South Asian than White samples.

### Principal Finding 2: References to the Heart

A 1996 study used the BSI to measure somatic symptoms among patients of different ethnicities in South London. The authors found that South Asian (predominantly Pakistani, some Indian) patients were more likely than White patients to report “weak or sinking heart” on the BSI.^42^ This was identified as potentially being related to idiomatic use of these phrases in both Punjabi and Urdu. In Punjabi, “sinking heart” refers to a condition that has both physiological and psychological aspects, which are understood to interrelate.^69^ “The heart sinks” is used in Urdu to express sadness and despair, while “weakening heart” can be used to describe someone who is easily fearful or overwhelmed. Similar language was used in qualitative studies. In a 1996 qualitative study of South Asian (mostly Punjabi) women in Britain, participants used expressions including “my heart kept falling and falling”; “the life would go out of my heart", “my heart is weak”; “my heart felt as if someone was pushing it down.”^28^ A 2012 qualitative study of Pakistani (85%) and Indian (15%) Muslim immigrants in Toronto, Canada, reported that Punjabi, Hindi and Urdu speaking participants described heart-related experiences including “sinking heart”, “dead heart”, and “emptiness of heart”.^24^

It is not possible to clearly delineate between references to the heart that refer to somatic symptoms, and those which do not. Indeed, there are different perspectives on whether references to the body in accounts of distress and depression should be understood as somatic, metaphorical, or as expressions of a way of understanding distress that understands the body and mind as part of a single experience. A 1995 quantitative study of elderly Bengali people in London found that 77% of participants reported heart-related symptoms such as pounding and breathlessness.^34^ In contrast, Akram, author of the aforementioned 2012 study of South Asian Muslims in Toronto, interpreted expressions relating to the heart as referring to affective, rather than somatic experiences, including sadness and emotional turmoil.^24^ However, quotes from participants in her thesis suggest that heart-related symptoms were at times experienced physically.

“I was feeling as if someone has held my heart in his hand tightly and squeezing it continuously … sometimes I was not able to breathe properly.” (p.113) (Pakistani Urdu speaker)

Some have suggested that references to the body, including the heart, in South Asian expressions of distress should be understood within the context of a non-Cartesian understanding of the relationship between mind and body. Krause argues that, for Punjabis, sinking heart “has at the same time a somatic and psychological meaning.”^69^ Participants in several studies made reference to cognitive symptoms in relation to the heart, using phrases including “thinking too much in my heart” (Punjabi participants),^28^ “my heart was sinking with these thoughts” (Indian Hindi speaker),^24^ and “thoughts and words in my heart” (South Asian Muslim).^51^ Participants in some papers made references to causal relationships between body and mind. For example, in a 1996 study of South Asian women in Britain, participants (who were mostly Punjabi) expressed that thinking in the heart led to illness, including pressure in the head (causing headaches).^28^ In a 2015 study on mental health conceptualisations among Nepalese young people in the UK, a participant expressed that “the worry or stress or tension keeps adding up and makes somebody have headaches or fever”.^60^ Meanwhile, a 1997 study comparing Pakistani depression patients in Britain to White British and Pakistani (in Pakistan) patients found that Pakistani patients living in Britain and in Pakistan understood depression through a conceptualisation of the “emotional body” (a term coined by Ots in relation to Chinese medicine),^70^ rejecting the body-mind dichotomy, and this distinguished them from White British patients.^43^ These accounts appear to support a non-dualistic conceptualisation of depression symptoms, with cognitive and somatic symptoms being experienced holistically. This is consistent with the Ayurveda and Unani Tibb medical systems, which are widely practiced in South Asian countries.^71,72^

### Principal Finding 3: Repetitive Negative Thinking

A 2006 cross-cultural comparison of conceptualisations of depression in the UK found that South Asian older adults were more likely to see depression as related to trouble with the mind and thinking, compared with Black Caribbean and White British older adults who were more likely to see it as related to mood.^54^ A 2007 comparison of British Pakistani and White British patients found Pakistani patients were more likely to refer to “too much thinking”.^32^ A 2017 cross-cultural comparison of experiences of depression in Australia found that people of Sri Lankan heritage explained their depression in terms of ‘overthinking’, while Anglo-Australians did not.^36^

Repetitive thinking was a reported symptom in five symptom analyses (Group A),^28,29,32,33,35^ and was also reported by participants in studies that did not provide an analysis of symptoms (Group C). Ways of describing this included “thinking negatively all the time"^24^, “thoughts go around in your head”^54^, “thinking too much”^28^, “thoughts repeating themselves “like a film”^28^, and “I repeatedly kept thinking about things”^29^. These experiences sometimes included references to a sense of thoughts being uncontrollable, and/or that repetitive thinking was having a detrimental impact on health.^33,60^ Repetitive negative thinking is not listed as a symptom of depression in ICD-11. One form of repetitive negative thinking is rumination,^73^ which has been found to be more common in depression where trauma is a factor, than in depression without trauma.^74,75^ Some studies reported depression symptoms as responses to trauma among Punjabi, Afghan, Tamil, and Indian participants.^27,44,53,55,56^ Meanwhile, trauma is known to be a common aspect of experience among migrant populations,^76,77^ and it has been argued that mental health among the South Asian diaspora is impacted by intergenerational trauma.^78^ It is possible that the prevalence of repetitive negative thinking among the South Asian diaspora may be related to trauma among this population.

### Gap Identified: Acculturation and Ethnic Differences

Only four papers considered acculturation differences in their analyses of depression symptoms.^31,37,39,43^ Each is at least 17 years old. A 2000 study assessed validity of the GHQ-12 and the ADI (developed in the Punjab region of India) among Punjabi and White English primary care patients in South London.^37^ The team found that the ADI was better at detecting depression among White English patients than Punjabi patients, but that it was not a valid tool for use with either group. Among Punjabi patients who had been in England for more than 30 years, the ADI was no better than chance. The GHQ-12 was a more valid tool for measuring depression in both Punjabi and White English patients, and the validity among Punjabi patients did not change depending on length of residency in England, which suggests there were no acculturation differences in validity of the GHQ-12. The article did not include analysis of acculturation differences in specific symptoms. A 2008 study of older South Asian Canadians (majority Indian) used the Geriatric Depression Scale (GDS) and found that length of residency was not significantly associated with depression caseness, but that those who had been in Canada for less than 10 years were more likely to report 'feeling not in good spirits most of the time' and 'not feeling happy most of the time' than those in Canada more than 10 years.^31^ It should be noted, however, that the GDS includes only one somatic symptom – psychomotor agitation. This means that none of the somatic symptoms most prevalent in South Asian countries^5^ were assessed. Two further studies found no acculturation differences.^39,43^ Overall, no acculturation pattern can be discerned from these four studies. There was a lack of literature on the specific experience of people of South Asian heritage born in English-speaking countries, and no papers compared this population to first-generation South Asian migrants.

No papers included in this review considered differences between people of different South Asian ethnicities. One study considered religious differences and found that Muslims reported ‘often get bored’ and ‘feel pretty worthless’ more often than Hindus and Sikhs, while there was no significant difference in overall depression prevalence between these groups.^31^ Overall, there was insufficient evidence to draw conclusions regarding effects of acculturation or ethnic differences in depression symptomology. This indicates a gap in research.

## Discussion

This systematic scoping review has found that the South Asian diaspora in English speaking countries experience symptoms of depression that differ from those of the White majority in those countries, and in ways that differ from the diagnostic criteria detailed in ICD-11. Physical pain (specifically headaches) and heart-related sensations are prevalent symptoms of depression in South Asian countries,^5^ and this review has found that these are also widely reported among diaspora populations. Sleep disturbances are commonly reported among South Asian diaspora populations and were found in more studies than anhedonia was, but studies directly comparing South Asian and White patients have not found sleep symptoms to be more prevalent in South Asian samples than White samples. Repetitive negative thinking may be more prevalent among South Asian diaspora groups than White majority groups in English speaking countries, which may be associated with trauma among South Asian migrant populations.^74,77–80^

Appropriate treatment requires accurate detection. Differences in symptoms of depression may therefore be implicative of inequalities in treatment, if those differences are not accounted for in diagnostic practice. Whilst ICD-11 has increased emphasis on cultural factors and diversity compared with previous versions, culturally informed diagnosis requires clinical judgement informed by cultural competence and cultural humility,^81^ rather than simple adherence to nosological critera.^82,83^ Despite attempts to recognise cultural differences in broad clinical guidelines^84,85^ greater attempts are needed within specific diagnostic classifications to recognise diverse experiences. Diagnosis in UK primary care relies on fast assessment of symptoms during short consultations, in a context of declining rates of continuity of care.^86^ Identification of culturally-specific patterns of symptomology is important both for the long-term goal of reducing nosological bias towards White populations in the Global North, and for improving diagnostic capacity among clinicians, including in primary care settings.

### Limitations and gaps

Findings are limited by the availability of literature. The review has found gaps in up-to-date literature on depression symptomology among South Asian diaspora populations, particularly in relation to acculturation differences, ethnic differences, and some South Asian ethnicities.

While 22 papers provided a synthesised list of symptoms of depression/distress or a comparison of symptoms across ethnicities, only three of these set out explicitly to describe symptoms of depression among a South Asian diaspora population.^22,30,31^ Other papers focussed on related questions such as experiences of migration,^24^ cultural representations of depression,^36^ depression prevalence,^39^ experiences of help seeking,^27^ prevalence of specific symptoms (such as somatic symptoms^41^ or self-harm^40^), and the cultural appropriateness of depression screening tools.^37^ This may mean that the lists of symptoms offered in such papers may not be comprehensive. This discrepancy in the aims and objectives of included papers, compared to the aim of the present review, may have caused bias in the findings available for review. Figure 2 and Table 2 should be read with consideration of this. The review has identified a shortage of research specifically aiming to describe depression symptoms among the South Asian diaspora.

Only ten reviewed studies were conducted within the last decade, so some are likely to be based on data that does not reflect the current socio-political environment. It was not possible to address the question of whether people of South Asian heritage who are born in English-speaking countries share symptomology with first-generation migrants or those living in South Asian countries. A qualitative study has found that GPs perceive acculturation differences in how South Asian patients present with chronic and unexplained pain, which indicates that acculturation differences may be important for understanding the role of pain in depression.^87^ Research is needed to explore differences between depression symptoms among first-generation South Asian migrants and people of South Asian heritage born in English-speaking countries.

As there are substantial cultural, religious, socio-economic, and political differences across the South Asian region, and migration patterns vary substantially, it is anticipated that the South Asian diaspora are unlikely to present as a homogenous group.^8^ Available literature is insufficient to support conclusive comparisons between ethnicities. Mood disorders have been found to be more prevalent among Pakistani Muslim women than Indian Hindu women in the UK, which indicates ethnic or religious differences in depression between these groups (which may relate to prevalence, detection, and/or help-seeking).^88^ Moreover, the majority of papers focussed on Punjabi, Indian, Pakistani, or unspecified South Asian populations, while some South Asian ethnicities, such as Nepalese, Bhutanese and Afghan, were not represented in any of the papers that provided analyses of symptoms.

### Implications for clinical practice and policy

This review suggests that GPs assessing South Asian diaspora patients should consider the possibility of non-ICD-11 somatic symptoms in diagnosis of depression, particularly physical pain. These may be reported by patients as the presenting symptom, rather than affective or cognitive symptoms.^61^ People of various South Asian communities use references to the heart when describing their symptoms of depression, and primary care practitioners should be alert to these references as potentially indicative of mood disorders. Members of South Asian diaspora populations also experience repetitive negative thinking as part of depression, possibly at higher rates than the White majority in English-speaking countries. This may be related to high levels of trauma within these populations.

These findings call into question the appropriateness of depression screening tools that are widely used in UK National Health Service (NHS) primary care settings and in NHS Talking Therapies, in patients of South Asian heritage. They support arguments for the need for clinical judgment informed by cultural humility in the application of nosology in context.^83^ Training of primary care professionals in specific presentations of depression among South Asian diaspora populations is therefore recommended.

### Statement from the PAPER Study Patient and Public Involvement Panel

We are a group of five, living in England, and of Indian and Pakistani heritage, all with experience of living with depression or supporting a loved one living with depression. Depression is under-recognised in our communities. Sometimes it manifests as physical symptoms, such as headaches, muscle tension, or chest pain. This highlights the significant interplay between mental health challenges and physical discomfort. In some cases, we use terms like “tension” or “worry” to describe ongoing psychological stress and distress, and we may not always understand how these experiences can relate to mental health diagnoses such as anxiety or depression. Older generations often find it harder to understand emotional struggles, which creates a generational divide in perceptions of mental health. This becomes more complex in immigrant families, and often emotional burdens are carried in silence, especially after difficult caregiving experiences. Our religious practices also have a direct impact on our perceptions of depression, and consequently on our help seeking behaviours. There is a need for improved understanding of mental health within South Asian communities, as well as the critical need for healthcare professionals to enhance their understanding of South Asian populations to provide tailored and culturally appropriate support that we need.

## Conclusion

This systematic scoping review has found that some symptoms of depression prevalent in South Asian countries are experienced by South Asian diaspora populations. Specifically, the review found evidence of people of South Asian heritage who live with depression experiencing sleep-related symptoms, physical pain and heart-related symptoms. Repetitive negative thinking was also identified as an important part of the experience of depression among South Asian diaspora groups, which could be related to trauma experiences among these populations. Neither physical pain, heart-related symptoms, nor repetitive negative thinking are detailed in ICD-11 diagnostic criteria for depressive disorders. Further research is needed to determine acculturation differences in symptomology, and differences between different ethnic groups within the South Asian diaspora. The findings of this review call into question the use of depression screening tools based on ICD-11 nosology among patients of South Asian heritage and point to the importance of cultural humility in clinical practice in primary care to reduce the potential for ethnic inequalities in treatment.

## Supplementary Material

Appendices

## Figures and Tables

**Table 1:** Comparing South Asian diaspora and White samples (Group B papers)

	Qualitative (Qual) /Quantitative (Quant)	Ethnicity (South Asian sample)	Sample size (South Asian sample)	Age (South Asian sample)	Sex (Female:Male) (South Asian sample)	Sample size (White sample)^1^	First generation (% of South Asian sample)	Country of residence	Screening tool(s) used	Comparative findings	Symptoms coded as potentially more important or prevalent in a South Asian diaspora population than a White populatin. (See Table 2)
**Antoniades, Mazza & Brijnath (2017)**	Qual	Sri Lankan	18	Mean=40	55:45	30	NR	Australia	K10^2^; DSM-IV^3^.	Symptom profiles similar and aligned with DSM. Somatisation not major theme in either group. Suicidal ideation less common in Sri Lankan participants. People of Sri Lankan heritage explained their depression in terms of **‘overthinking’**, while Anglo-Australians did not.	Repetitive and/or negative thinking
**Bhui, Bhugra & Goldberg (2000)**	Quant	Punjabi	209	Mean=46	NR	185	84	UK	ADI^4^; GHQ-12^5^	Study compared which items across two screening tools are case predictors of depression for Punjabi and White participants. Items that were depression case predictors for Punjabi participants included those that indicated anhedonia, changes in sleep, worthlessness, difficulty concentrating, low mood, and stress Items that were case predictors for White participants included those that indicated all these symptoms *except*** difficulty concentrating**.	Difficulty concentrating
**Bhui et al.** **(2001)**	Quant	Punjabi	209	Mean=46	NR	185	84	UK	ADI; GHQ-12	Punjabi patients more likely to have **poor concentration and memory, “depressive ideas”, **and **physical pain. **No ethnic difference in prevalence of somatic symptoms overall. Punjabi patients less likely to acknowledge emotional cause of physical symptoms.	Difficulty concentrating Repetitive and/or negative thinking Physical pain
**Bhui et al.** **(2004)**	Quant	Punjabi	209	Mean=46	NR	185	84	UK	ADI; GHQ-12	Punjabi patients with somatic symptoms (defined as **“any sort of ache or pain, for example headache or indigestion, or any other sort of bodily discomfort" that the patient considers is "due to, or made worse by, feeling low, anxious or stresse**d") were more likely to be depressed than White patients with somatic symptoms.	Physical pain
**Brijnath & Antoniades (2018)**	Qual	Indian	28	Range=19 -84	54:46	30	NR	Australia	K10	Both groups reported a sense of heaviness associated with depression. Language used by Indian participants, which was not used by White participants, included **“harder to get out of bed, harder to do my work”, “something squashing you inside”, someone should wrap me up in cotton wool”. **Indian participants more likely to see depression as a short-term ailment, while White participants more likely to see it as long term, but when Indian participants did not get better quickly, this led to **hopelessness.**	Hopelessness Overwhelm, unable to function Physical pressure (feeling of) Fragility (feeling of)
**Cooper et al.** **(2006)**	Quant	South Asian	220	16- 24=65%; 25- 34=20%; 35- 64=15%	75:25	3,574	NR	UK	N/A. Findings based on medical records.	Study of patients admitted to hospital for self-inflicted injuries. Assessed depression using 7 items: Feeling depressed; Looks depressed; Feeling hopeless; Suicidal plans; Suicidal thoughts; Sleep problems; Appetite problems. All items recorded at higher rate in in White patients than South Asian patients, so South Asian patients less likely to be assessed as depressed.	None
**Farooq et al.** **(1995)**	Quant	Asian (predominantly Indian and Pakistani)	87	Mean=34	47:53	108	82	UK	BSI^6^; HADS^7^.	Positive correlations between BSI scores and HADS depression and anxiety scores. This correlation was similar in both ethnic groups. Anxiety more strongly correlated with somatic symptoms than depression was, for both groups.	None
**Malik (1997)**	Quant	Pakistani	120	Range=25 -60	50:50	120	100	UK	GHQ; AKAUDS^8^	Pakistani participants reported more somatic symptoms than White participants on GHQ and AKAUDS (items reporting **feeling run down, experiencing pain and pressure in the head, temperature related sensations, and loss of sleep**). In interviews, most prevalent symptoms reported by Pakistani participants were affective. Biggest difference between Pakistani and White participants was that Pakistani participants' affective descriptions closely tied to relationships, while White participants descriptions were more individualised.	Temperature related sensations Physical pain: headache Physical pressure (feeling of) Changes in sleep Fatigue
**Mallinson &** **Popay (2007)**	Qual	Pakistani	31	Range=19 -65	52:48	27	61	UK	SRQ^9^; SCAN^10^; LEDS^11 ^used to identify participants. Findings of study are based on qualitative interviewing	Both groups described low mood, stress, crying, anger, fatigue, numbness, frustration, low self-esteem, and hopelessness. lethargy and sleeplessness. Pakistani participants spoke about **temperature changes**, which White participants did not. Both groups used metaphors of drowning. Pakistani participants described **"pressure in the head", "too much thinking", "heavy head", and mental tension. **Pakistani participants were more likely to make links between body and mind. Pakistani participants did not discuss suicidal feelings as much as White participants, although four did mention it briefly. White participants were more willing to discuss suicidality in more detail.	Physical pain: headache Physical pressure (feeling of) Heaviness (feeling of) Temperature-related sensations
**Mansour et al. (2020)**	Quant	South Asian	166	Range=65 +	64:36	3,931	NR	UK	N/A. Findings based on medical records.	South Asian patients were less likely to present with substance use problems, guilt feelings, hopelessness or suicidal thoughts, compared to White British patients. No significant difference recorded in somatic symptoms.	None
**Sham et al.** **(1996)**	Quant	Asian (predominantly Pakistani, also Indian)	87	Mean=34	47:53	108	84	UK	HADS; BSI	Emotional item scores not substantially different between ethnic groups, but somatic item scores were. **"Weak or sinking heart" "Aches and pains all over the body" and "Feeling of heat inside your body" **were predictors of Asian ethnicity. These all relate to Urdu or Punjabi idioms.	Heart-related experiences Pain: aches and pains Temperature related sensations

1 White and South Asian samples were not matched on sex or age.

2 Kessler Psychological Distress Scale

3 Diagnostic and Statistical Manual of Mental Disorders

4 Amritsar Depression Inventory

5 General Health Questionnaire

6 Bradford Somatic Index

7 Hospital Anxiety and Depression Scale

8 Aga Khan University Anxiety and Depression Scale

9 Self-Reporting Questionnaire

10 Schedules for Clinical Assessment in Neuropsychiatry

11 Life Events and Difficulties Schedule

**Table 2:** All reported symptoms

	Symptom	Papers in which symptom featured in analytic findings, and/or in reference to individuals/participant quotation	Reported in analysis of all symptoms in a South Asian diaspora sample in one or more paper	Identified as more important or prevalent in a South Asian diaspora population than a White population in one or more paper	Reported in both qualitative and quantitative analytic findings
**ICD-11**	Anhedonia	24, 25, 27, 28, 31-34, 36, 37, 45, 47, 48, 51	✓		✓
	Low mood	23-34, 36, 37, 47, 49, 50, 56, 58, 60	✓		✓
	Changes in appetite	26- 28, 30, 32, 33, 45, 47, 54-56, 59, 60	✓		
	Changes in sleep	23, 26- 28, 30, 32, 33, 35, 37, 43, 44, 46, 49, 54-57, 60, 61	✓	✓	✓
	Difficulty concentrating	24-26, 32, 33, 36- 38, 56, 60	✓	✓	✓
	Fatigue	26, 28, 30, 32, 33, 35, 36, 43, 45, 46, 48, 51, 53, 56-59	✓	✓	✓
	Hopelessness	24-26, 29, 30, 32, 33, 35, 36, 49, 53, 55, 59	✓	✓	✓
	Psychomotor retardation or agitation	31, 32, 47	✓		✓
	Recurrent thoughts of death or suicide	28, 30, 32, 36, 48, 49, 52, 53, 55, 59, 61	✓		✓
	Worthlessness, guilt	24, 28, 30, 32, 35, 37, 48, 49, 53- 55, 58-60	✓		✓
**Non-ICD-11**	Anger or irritability	25, 30, 32, 33, 35, 50, 55-57	✓		
	Boredom	31, 33	✓		
	Breathing difficulties	26, 49	✓		
	Cold sores*	60			
	Confusion forgetfulness indecisiveness	24, 25, 31, 33, 35, 38, 56, 58	✓		✓
	Crying	26-28, 30-33, 46, 48-50, 52, 53, 56	✓		✓
	Digestive symptoms	26, 30, 44	✓		
	Dissociation or detachment*	26, 49			
	Fainting, dizziness*	24, 49, 56			
	Fragility (feeling of)	25		✓	
	Hair loss*	45			
	Heart related experiences	24, 26, 28, 34, 42, 47, 51	✓	✓	✓
	Heaviness (feeling of)	25, 32, 47	✓	✓	
	Helpless/powerless/trapped (feeling of)	26, 29, 30, 33, 35, 36, 49, 50, 52, 53	✓		✓
	Loneliness/isolation (feeling of)	33, 43, 45, 49, 50, 52, 55, 59	✓		✓
	Nightmares*	49			
	Numbness	32	✓		
	Overwhelm/inability to function	24, 25, 28, 35, 51, 53, 54, 56, 59	✓	✓	
	Physical pain	24, 26, 28, 30, 32-35, 38, 39, 42, 43, 45, 46, 51, 56, 59-61	✓	✓	✓
	Physical pressure (feeling of)	25, 26, 28, 29, 32, 43, 49	✓	✓	✓
	Pins and needles*	49, 56			
	Repetitive and/or negative thinking	24, 28-30, 32, 33, 36, 38, 44, 51, 54, 58, 60	✓	✓	✓
	Self-harm*	52			
	Self-neglect*	47, 53, 55, 56			
	Self-pity*	50			
	Social withdrawal	27, 29, 33, 49, 53, 57, 58, 60	✓		
	Stress or under strain	32, 37, 44, 47, 49, 50, 56	✓		✓
	Substance abuse	35, 52, 55, 56	✓		
	Temperature related sensations	28, 32, 42, 43, 49	✓	✓	✓
	Visual disturbance*	49			

* Symptom mentioned only in description of individuals or in participant quotation. Not included in any paper’s reported analytic findings.

## Data Availability

A summary of the charted data is included as an appendix. A full copy of the charted data is available on request from the corresponding author (RR).

## Footnotes

i People of Afghan, Bangladeshi, Bhutanese, Indian, Maldivian, Nepalese, Pakistani, or Sri Lankan heritage.

ii Countries were identified as having large South Asian diasporas if a) more than 3% of their population is of South Asian heritage; or b) the number of people of South Asian heritage living in the country exceeds 1 million.

iii Excessive worry and psychosis were excluded, as these are indicators of alternative psychiatric diagnoses.

iv For quantitative studies focussed on a population with depression or distress symptoms, symptoms were coded if they were experienced by more than 50% of participants. For quantitative studies focussed on a general population, the most common two symptoms found in the population were coded. Full details of reported symptoms and their prevalence can be found in the table of charted data supplied inAppendix 3.

v Some studies identified participants according to national heritage (e.g. Indian), while others identified them according to ethnic or regional heritage (e.g. Punjabi; Tamil, Bengali).

## Analytic Code Availability

Qualitative codes used in the thematic analysis presented are available on request from the corresponding author (RR).
