# Supplementary material for: Understanding Depression Symptom Heterogeneity in South Asian Minority Groups: A Systematic Scoping Review
Source: Br J Psychiatry. Author manuscript; Available in PMC 2026 Mar 16. (PMC7618885; doi:10.1192/bjp.2026.10539)
Supplement: Appendices [file EMS211971-supplement-Appendices.pdf]

Appendices

Appendix 1: Search Strategy

<table>
<tr><th colspan="3">Inclusion Criteria</th></tr>
<tr><td colspan="2">Participants/Sample</td><td>Individuals from the South Asian diaspora (Limited to 18+)</td></tr>
<tr><td colspan="2">Concept</td><td>Experiences of depression</td></tr>
<tr><td colspan="2">Context</td><td>English speaking countries with large South Asian diaspora populations</td></tr>
<tr><td colspan="2">Type of evidence sources</td><td>Quantitative, qualitative and mixed-methods primary research studies. Grey literature including local NHS trust reports and guides, government reports and briefings, and educational theses</td></tr>
<tr><th colspan="3">Exclusion Criteria</th></tr>
<tr><td colspan="2">Participants/Sample</td><td>Samples which include those outside of South Asian diaspora where findings from South Asian individuals cannot be isolated</td></tr>
<tr><td colspan="2">Concept</td><td>Comorbidity research where depression symptoms have been considered alongside another condition e.g., depression with physical condition. Research focuses solely on postpartum depression.</td></tr>
<tr><td colspan="2">Language</td><td>Sources not published in English</td></tr>
<tr><td colspan="2">Analysis</td><td>Quantitative research that does not report symptoms on an item-by-item level</td></tr>
<tr><th colspan="3">Identification of Sources</th></tr>
<tr><td rowspan="4">Key words</td><td>Concept</td><td>Search Terms</td></tr>
<tr><td>South Asian Diaspora</td><td>South Asian: south asia* / ethnic* minorit*/ BME / BAME / Bangladesh* / india* / sri lanka* / Pakistan* / Nepal* / Maldives*/ Bhutan*</td></tr>
<tr><td>Depression</td><td>Depression: depress*/ “low mood”/ distress/ depressive disorder/ melancholia/ “mood disorder”/ “affective disorder”/ affective symptoms/ major depress/ dysphori*</td></tr>
<tr><td>English Speaking Countries with Large South Asian Diaspora Populations</td><td>United Kingdom/Great Britain/Channel Islands/England/Northern Ireland/Scotland/ Wales.
United States / Alabama / Alaska / Arizona / Arkansas / California / Colorado / Connecticut / Delaware / Florida / Georgia / Hawaii / Idaho / Illinois / Indiana / Iowa / Kansas / Kentucky / Louisiana / Maine / Maryland / Massachusetts / Michigan / Minnesota / Mississippi / Missouri / Montana / Nebraska / Nevada / “New Hampshire” / “New Jersey” / “New Mexico” / “New York” / “North Carolina” / “North Dakota” / Ohio / Oklahoma / Oregon / Pennsylvania / “Rhode Island” / “South Carolina” / “South Dakota” / Tennessee / Texas / Utah / Vermont / Virginia / Washington / “West Virginia” / Wisconsin / Wyoming
Canad*/ Alberta/ “British Columbia”/Manitoba/ “New Brunswick”/ Newfoundland/ “Labrador”/ “Northwest Territories”/ “Nova Scotia”/ Nunavut/ Ontario/ Prince Edward Island/ Quebec/ Saskatchewan/ Yukon
Australia: Australia*/“New South Wales”/“Northern Territory”/ “Queensland”/ Victoria*/ “Western Australia”/ “South Australia”
New Zealand: New Zealand/ “Bay of Plenty”/ Canterbury/ Gisborne/ Hawke’s Bay/ Marlborough/ Nelson/ Northland/ Otago/ Southland/ Taranaki/ Waikato/ Wellington/ “West Coast”/ Whanganui</td></tr>
<tr><td colspan="2">Databases</td><td>SCOPUS, EMBASE, PubMed, PsycINFO, Web of Science, Google Scholar, Kings Fund</td></tr>
<tr><td colspan="2">Manual sources and hand searching</td><td>Include records identified through other sources and through hand searching. Forward and backward citation search of identified sources.</td></tr>
</table>

Appendix 2: Data charting form

- Citation Details
- Source type
- Research Design and Context
    - Aim
    - Country
    - Qualitative/Quantitative
    - Sampling strategy
    - Inclusion and exclusion criteria
    - Recruitment
    - Sample size (South Asian sample)
    - Data collection method
    - Screening tool (if used)
    - Analytic method
    - Did the paper include a comprehensive analysis of symptoms, providing a list of symptoms drawn from the whole dataset? Yes/No
        - Analysis based on ICD/DSM?
    - Comparison between ethnicities?
- Sample
    - Ethnicity
    - % First generation migrants
    - Sex
    - Age
    - Religion
    - Language
    - Diagnosis of or treatment for depression?
- Reported symptoms
    - ICD-11 symptoms
    - Non-ICD-11 symptoms
    - Differences in symptoms between South Asian participants and other participants
    - Duration of symptoms
    - Prevalence of symptoms
    - Acculturation differences
    - Generational differences
    - Differences between different South Asian ethnicities
    - Other demographic differences

Appendix 3: Summary of charted data

Author (Year)	Source type	Qualitative / Quantitative	Population of focus	South Asian Sample	Reported symptoms	Classification for review (Group A/B/C)
Agarwal-Narale (2005)	Thesis (MA)	Qualitative	South Asian immigrant women in Canada	N=9<br>Ethnicity: Indian (33.3%); Pakistani (66.6%).<br>First generation migrants: 100%<br>Female:Male ratio: 100:0<br>Age range=25-44	Difficulty sleeping, digestion problems, "in stress", "tension"	C
Ahmad et al. (2005)	Journal article	Qualitative	Indian immigrant women in Canada	N=24<br>Indian (100%)<br>First generation migrants: 100%<br>Female:Male ratio: 100:0<br>Age: range=18-69; mean=34	Loneliness, loss of appetite, anhedonia, headaches, back pain, joint pain, fatigue, hair loss.	C
Ahmed et al. (2017)	Journal article	Quantitative	Muslim South Asian women in UK	N=50<br>Ethnicity: South Asian (100%)<br>Female:Male ratio: 100:0<br>Age: 66% <45; 34% >45<br>Religion: Muslim (100%)	Exact percentages not reported.<br>Over 60% reported low mood.<br>Nearly 60% reported sleep disturbance.<br>Over 40% reported difficulty concentrating.<br>Over 40% reported difficulty relaxing.	A
Akram, S. (2012)	Thesis (Doctor of Education)	Qualitative	South Asian immigrant women in Canada who have experienced and recovered from depression since migrating.	N=13<br>Ethnicity: Pakistani (85%); Indian (15%)<br>First generation migrants: 100%<br>Female:Male ratio: 64:36<br>Age: range=19-53; mean=38<br>Religion: Muslim (100%)	Physical pain; "emotional turmoil" (often expressed through heart related imagery); anhedonia; concentration and memory difficulties; worthlessness; sadness; hopelessness.	A

**Author (Year)**	**Source type**	**Qualitative / Quantitative**	**Population of focus**	**South Asian Sample**	**Reported symptoms**	**Classification for review (Group A/B/C)**
Antoniades, Mazza, & Brijnath (2017)	Journal article	Qualitative	Sri Lankan and Anglo-Australian people living with depression (clinically diagnosed or self-identified).	N=18<br>Ethnicity: Sri-Lankan (100%)<br>Female:Male ratio: 55:45<br>Age: range=18-78; mean=40<br>Religion: Christian (28%); Hindu (22%); Buddhist (22%); Atheist/Agnostic (22%); Other (6%)	Symptom profiles similar in Sri Lankan and Anglo-Australian samples and aligned with DSM. People of Sri Lankan heritage explained their depression in terms of 'overthinking', while Anglo-Australians did not.	B
Arneja (2013)	Thesis	Qualitative	South Asian migrant women in California, USA.	N=5<br>Ethnicity: Indian (60%); Pakistani (40%)<br>First generation migrants: 100%<br>Female:Male ratio: 100:0<br>Age: range=48-60<br>Religion: Sikh (60%); Muslim (40%)	Fatigue; crying; sleep disturbance; general aches and pains.	C
Bhui, Bhugra, & Goldberg, (2000)	Journal article	Quantitative	Punjabi and White English primary care patients, London, UK	N=209<br>Ethnicity: Punjabi (100%)<br>First generation migrants: 84%<br>Age: mean=46<br>Religion: Sikh (85%); Muslim (8%); Hindu (6%); Christian (0.5%)	Measured which items on ADI and GHQ-12 were case predictors (CIS-R 11/12) for depression. 8/42 items across the two tools were case predictors for Punjabi patients. (ADI: I feel unhappy most of the time. I am unable to fully commit myself to anything; I feel that I am inferior to others. GHQ-12: Lost much sleep over worry; Felt constantly under strain; Able to concentrate on what you're doing; Been feeling unhappy or depressed; Feeling reasonably happy.	B
Bhui et al. (2001)	Journal article	Quantitative	Punjabi and White English primary care patients, London, UK	N=209<br>Ethnicity:Punjabi (100%)<br>First generation migrants: 84%<br>Age: mean=46<br>Religion: Sikh (85%); Muslim (8%); Hindu (6%); Christian (0.5%)	Punjabi patients more likely to have poor concentration and memory, "depressive ideas", and physical pain. No ethnic difference in prevalence of somatic symptoms overall.	B

**Author (Year)**	**Source type**	**Qualitative / Quantitative**	**Population of focus**	**South Asian Sample**	**Reported symptoms**	**Classification for review (Group A/B/C)**
Bhui et al. (2004)	Journal article	Quantitative	Punjabi and White English primary care patients, London, UK	N=209<br>Ethnicity: Punjabi (100%)<br>First generation migrants: 84%<br>Age: mean=46<br>Religion: Sikh (85%); Muslim (8%); Hindu (6%); Christian (0.5%)	Punjabi patients with somatic symptoms (defined as "any sort of ache or pain, for example headache or indigestion, or any other sort of bodily discomfort" that the patient considers is "due to, or made worse by, feeling low, anxious or stressed" were more likely to be depressed than White patients with somatic symptoms.	B
Bottorff et al. (2001)	Journal article	Qualitative	South Asian women, Western Canada	N=80<br>Ethnicity: South Asian<br>First generation migrants: 100%<br>Female:Male ratio: 100:0<br>Religion: Sikh (61%); Muslim (18%); Hindu (12%); Christian (4%)	Participants referred to their experience as "stress". Symptoms included loss of appetite, anhedonia, psychomotor retardation, lack of self-care.	C
Brijnath & Antoniades (2018)	Journal article	Qualitative	Anglo-Australian or Indian Australian, adults, diagnosed with depression, UK	N=28<br>Ethnicity: Indian (100%)<br>Age: range=19-84<br>Female:Male ratio: 54:46	Sadness; difficulty concentrating; forgetfulness; apathy; agitation; a sense of 'heaviness'.	A & B
Burr (2002)	Journal article	Qualitative	South Asian women who felt they had experienced feelings of depression, UK	N=46, including focus groups and interviews. Demographic data provided only for interview participants, n=10.<br>Ethnicity: Pakistani (50%); unspecified "Indian subcontinent" (50%)<br>First generation migrants: 60%<br>Age: range=21-61; mean=37<br>Female:Male ratio: 100:0<br>Religion: Muslim (60%); Hindu (20%); Sikh (20%)	Low mood; powerlessness; hopelessness; detachment; crying; nausea and vomiting; general aches and pains; headaches; period pain; asthma attacks.	A

**Author (Year)**	**Source type**	**Qualitative / Quantitative**	**Population of focus**	**South Asian Sample**	**Reported symptoms**	**Classification for review (Group A/B/C)**
Chalal (2018)	Thesis	Qualitative	Punjabi people who have lived with depression or have a family member who has lived with depression, Canada	N=6<br>Ethnicity: Punjabi (100%)<br>First generation migrants: 80%<br>Age: range=28-56; mean=43<br>Female:Male ratio: 83:17	Low mood; changes in sleep; changes in appetite; anhedonia; social withdrawal.	A
Conrad & Pacquiao (2005)	Journal article	Qualitative	Indian patients admitted with depression to acute psychiatric hospital, USA	N=20<br>Ethnicity: Indian (100%)	Crying; worthlessness; purposelessness; suicidal ideation; fatigue.	C
Cooper et al. (2006)	Journal article	Quantitative	Self-harm attendees to Accident and Emergency, comparing South Asian patients with White patients, Manchester and Salford, UK	N=220<br>Age: 16-24=65%; 25-34=20%; 35-64=15%<br>Female:Male ratio: 78:22	Study of patients admitted to hospital for self-inflicted injuries. Assessed depression using 7 items: Feeling depressed; Looks depressed; Feeling hopeless; Suicidal plans; Suicidal thoughts; Sleep problems; Appetite problems. All items recorded at higher rate in in White patients than South Asian patients, so South Asian patients less likely to be assessed as depressed.	B
Dein (2013)	Book chapter	Qualitative	Bangladeshi Muslims, London, UK	N=30<br>Ethnicity:Bangladeshi (100%)<br>Religion: Muslim (100%)	Low mood; hopelessness; worthlessness; sleep disturbance; nightmares; worry; stress; dizziness; breathlessness; visual disturbance; pins and needles; heat sensation; feeling of isolation; disassociation; feeling that everything is moving; crying; suicidal ideation; "weakness of mind", "a presence trying to attack him and suffocate him at night".	C

**Author (Year)**	**Source type**	**Qualitative / Quantitative**	**Population of focus**	**South Asian Sample**	**Reported symptoms**	**Classification for review (Group A/B/C)**
Farooq et al. (1995)	Journal article	Quantitative	Asian and White primary care attendees, UK	N=87<br>Ethnicity: "Asian", predominantly Indian and Pakistani<br>First generation migrants: 82%<br>Age: mean=34 (SD=15.6)<br>Female:Male ratio: 47:53	Positive correlations between BSI scores and HADS depression and anxiety scores. This correlation was similar in both ethnic groups. Anxiety more strongly correlated with somatic symptoms than depression was, for both groups.	B
Fenton et al. (1996)	Journal article	Qualitative	South Asian women, including those who were identified by community workers as having "life difficulties", Bristol, UK	N=59<br>Ethnicity: South Asian, majority Punjabi<br>First generation migrants: 98%<br>Age: Early 20s- early 60s<br>Female:Male ratio: 100:0<br>Religion: Muslim (65%); Sikh (35%)	Loss of purpose; worthlessness; inability to cope with daily tasks; loss of appetite; fatigue; suicidal ideation; sleep disturbance; tearfulness; body aches and pains.	A
Gask et al. (2011)	Journal article	Qualitative	Pakistani women being treated for depression, Lancashire, UK.	N=15<br>Ethnicity: Pakistani (100%)<br>First generation migrants: 73%<br>Age: range=23-73; mean=42<br>Female:Male ratio: 100:0	Low mood; feeling stuck; social withdrawal; sense of lack of control.	A
Grewal (2009)	Thesis (MA)	Qualitative	South Asian Sikhs who self-identified as having feelings of depression, Canada	N=6<br>Ethnicity: Punjabi (100%)<br>First generation migrants: 33%<br>Age: range=20-40; mean=28<br>Female:Male ratio: 83:17<br>Religion: Sikh (100%)	Hopelessness; worthlessness; sadness; sleep disturbance; anhedonia; apathy; fatigue; changes in appetite; suicidal thoughts; irritability; crying; physical pain; racing thoughts; weight loss; vomiting; paranoia; anxiety; feeling powerless.	A
Hashwani (2006)	Thesis (PhD)	Qualitative	First generation Pakistani immigrants, California, USA	N=24<br>Ethnicity: Pakistani (100%)<br>First generation migrants: 100%<br>Age: range=21-77<br>Female:Male ratio: 54:46	Crying; loneliness; self-pity; stress; irritability; low mood.	C

**Author (Year)**	**Source type**	**Qualitative / Quantitative**	**Population of focus**	**South Asian Sample**	**Reported symptoms**	**Classification for review (Group A/B/C)**
Husain, Creed & Tomenson (1997)	Journal article	Quantitative	Primary care attendees of Pakistani heritage aged 16-64, UK. All were invited to complete PHQ. All high scorers and a random selection of low scorers invited for interview. Study based on quantitative interview data.	N=77<br>Ethnicity: Pakistani (100%)<br>First generation migrants: 77%<br>Age: range=22-44<br>Female:Male ratio: 56:21	Thoughts of self-harm and suicide; physical pain; sleep disturbance.<br>44 patients identified as depressed using PHQ. Only one of these had been diagnosed with depression by GP. For the remaining 43, GP notes mentioned physical pain related symptoms, but these were also mentioned for patients who were not identified as depressed using PHQ.	C
Hussain & Cochrane (2002)	Journal article	Qualitative	Women identified as being from Indian subcontinent, who had received treatment from mental health services for clinical depression within the past year, UK.	N=10<br>Ethnicity: South Asian<br>First generation migrants: 70%<br>Female:Male ratio: 100:0<br>Religion: Muslim (60%); Hindu (30%); Sikh (10%)	"Couldn't get out of bed"; headaches; fatigue; "thoughts in my heart"	C
Hussain & Cochrane (2003)	Journal article	Qualitative	Women identified as being from Indian subcontinent, who had received treatment from mental health services for clinical depression within the past year, UK.	N=10<br>Ethnicity: South Asian<br>First generation migrants: 70%<br>Female:Male ratio: 100:0<br>Religion: Muslim (60%); Hindu (30%); Sikh (10%)	Crying; self-harm; substance abuse; loneliness; helplessness	C

**Author (Year)**	**Source type**	**Qualitative / Quantitative**	**Population of focus**	**South Asian Sample**	**Reported symptoms**	**Classification for review (Group A/B/C)**
Johnson (2019)	Thesis	Qualitative	Patients of Indian heritage who completed NHS talking therapies through Improving Access to Psychological Therapies (IAPTS) within previous four weeks, West Midlands, UK.	N=7<br>Ethnicity: Indian (100%)<br>Age: range=22-65; mean=43<br>Female:Male ratio: 71:29	Tearfulness; hopelessness; low self-esteem; worthlessness, fatigue; self-neglect; sense of lack of control; suicidal ideation; social withdrawal.	C

**Author (Year)**	**Source type**	**Qualitative / Quantitative**	**Population of focus**	**South Asian Sample**	**Reported symptoms**	**Classification for review (Group A/B/C)**
Lai & Surood (2008)	Journal article	Quantitative	South Asians older adults, Canada,	N=210<br>Ethnicity: Indian (76%); Pakistani (9%); African South Asian (9%); Unreported (6%)<br>First generation migrants: 99%<br>Age: range=55-93; mean=66<br>Female:Male ratio: 44:56<br>Religion: Sikh (55%); Hindu (21%); Muslim (21%); Catholic (1%); Other (2%); None (0.5%)	Among those who were identified as depressed (n=45) using adapted validated version of Geriatric Depression Scale:<br><br>Basically not satisfied with life: 28.9%<br>Dropped many activities and interests: 71.1%<br>Feel that life is empty: 42%<br>Often get bored: 75.6%<br>Not in good spirits most of the time: 53.3%<br>Afraid that something bad is going to happen: 53.3%<br>Not feel happy most of the time: 42.2%<br>Often get restless and fidgety: 55.6%<br>Frequently worry about the future: 71.1%<br>Have more problems with memory: 53.3%<br>Often feel downhearted and blue: 51.1%<br>Feel pretty worthless: 46.7%<br>Think that most people are better off: 46.7%<br>Frequently get upset over little things: 53.3%<br>Frequently feel like crying: 55.6%	A

Author (Year)	Source type	Qualitative / Quantitative	Population of focus	South Asian Sample	Reported symptoms	Classification for review (Group A/B/C)
Lawrence et al. (2006)	Journal article	Qualitative	White British, South Asian, and Black Caribbean older adults, UK	N=33<br>Ethnicity: Indian (60%); Pakistan (9%); African South Asian (21%); Other (3%)<br>Age: ≥65<br>Female:Male ratio: 73:27<br>Religion: Hindu (60%); Muslim (15%); Sikh (15%); Christian (9%)	Worry; loss of confidence; unable to function; loss of appetite; sleep disturbance; recurrent or persistent thoughts.	C
Malik (1997)	Thesis	Quantitative	Pakistani and White British people, UK	N=120<br>Ethnicity: Pakistani (100%)<br>First generation migrants: 100%<br>Age: range=25-60<br>Female:Male ratio: 50:50	Pakistani participants reported more somatic symptoms than White participants on GHQ. No significant difference in somatic symptoms on AKAUDS, but somatic symptoms such as temperature-related sensations, headaches, and feeling of pressure in the head were endorsed more frequently in the British Pakistani sample than in the White British sample. In interviews, most prevalent symptoms reported by Pakistani participants were affective. Biggest difference between Pakistani and White participants was that Pakistani participants' affective descriptions closely tied to relationships, while White participants descriptions were more individualised. The most commonly endorsed items by British Pakstani participants, (excluding worry and anxiety), related to headaches and feeling lonely (AKAUDS), and fatigue and headaches (GHQ).	A&B

**Author (Year)**	**Source type**	**Qualitative / Quantitative**	**Population of focus**	**South Asian Sample**	**Reported symptoms**	**Classification for review (Group A/B/C)**
Mallinson & Popay (2007)	Journal article	Qualitative	People who had been identified as depressed or distressed using three screening tools used in wider epidemiological study, and who were White or Pakistani, aged 18-65, UK	N=31<br>Ethnicity: Pakistani (100%)<br>First generation migrants: 61%<br>Age: range=19-65<br>Female:Male ratio: 52:48	Fatigue; sleep problems; low mood; worthlessness; hopelessness; temperature changes; numbness (affective); anger; crying. Pakistani participants did not discuss suicidal feelings as much as White participants, although 4 did mention it briefly.	A & B
Mansour et al. (2020)	Journal article	Quantitative	Patients diagnosed with late life depression 2006-2017, treated at a mental health care provider, London, UK	N=166<br>Ethnicity: South Asian<br>Age: ≥65<br>Female:Male ratio: 64:36	South Asian patients were less likely to present with substance use problems, guilt feelings, hopelessness or suicidal thoughts, compared to White British patients. No significant difference recorded in somatic symptoms.	B
Morrow & Krishna (2019)	Journal article	Qualitative	Single case study of an Afghan refugee admitted to hospital following a suicide attempt, USA	N=1<br>Ethnicity = Afghan (100%)<br>First generation migrant: 100%<br>Age: 31<br>Female:Male ratio: 0:100	Suicide attempt; suicidal ideation; self-neglect; loss of appetite; insomnia; worthlessness; guilt; sense of alienation; anger; remorse; despair; lack of identity; hopelessness; alcohol abuse.	C
Pandalangat (2012)	Thesis (PhD)	Qualitative	Sri Lankan Tamils diagnosed with depression, Canada	N=16<br>Ethnicity: Sri Lankan Tamil (100%)<br>First generation migrants: 100%<br>Age: range=26-63; mean=41<br>Female:Male ratio: 50:50	Aches and pains; headaches; numbing (physical); sleep disturbance; forgetfulness; anger; change in appetite; fainting; alcohol abuse; stress; crying; fatigue; low mood; self-neglect.	C

**Author (Year)**	**Source type**	**Qualitative / Quantitative**	**Population of focus**	**South Asian Sample**	**Reported symptoms**	**Classification for review (Group A/B/C)**
Rafique (2010)	Journal article	Qualitative	Pakistani women with symptoms consistent with depression as per ICD-10, UK	N=7<br>First generation migrants: 57%<br>Age: range=24-48; mean=36<br>Female:Male ratio: 100:0<br>Religion: Muslim (100%)	Low mood; loss of interest; fatigue; hopelessness; sleep disturbance; change in appetite; concentration difficulties; aches and pains; headaches; repetitive negative thinking; forgetfulness; difficulties with decision-making; loneliness; social withdrawal; crying; feeling trapped.	A
Robinson (2016)	Thesis	Qualitative	Black and minority ethnic women with a depression diagnosis, UK.	N=2<br>Ethnicity: Bangladeshi (50%); Sri Lankan (50%)<br>First generation migrants: 50%<br>Age: 64 and 34<br>Female:Male ratio: 100:0	Excess sleep; self-isolation; anger; fatigue	C
Sembhi & Dein (1998)	Journal article	Qualitative	Case studies of psychiatric patients of Asian heritage treated in two mental health teams, UK.	N=3<br>Ethnicity: Pakistani (33%); Indian (33%); Unspecified Asian Muslim (33%)<br>First generation migrants: 67%<br>Age: 35; 46; 70<br>Female:Male ratio: 33:67<br>Religion: Muslim (67%); Sikh (33%)	Low mood; fatigue; negative thinking; forgetfulness; loss of confidence; social withdrawal	C
Shafique (2014)	Report	Mixed methods	Asian women in Oxfordshire, UK. Single case presented of one woman with depression.	N=1<br>Ethnicity: Bangladeshi (100%)<br>First generation migrants: 100%<br>Age: 36<br>Female:Male ratio: 100:0	Headache; fatigue; loss of appetite; worthlessness; suicidal ideation	C

Author (Year)	Source type	Qualitative / Quantitative	Population of focus	South Asian Sample	Reported symptoms	Classification for review (Group A/B/C)
Sham et al (1996)	Journal article	Quantitative	White British and Asian primary care attenders in an inner city and an outer city ward, UK	N=87<br>Ethnicity: South Asian (predominantly Pakistani, also Indian)<br>First generation migrants: 84%<br>Age: mean=34<br>Female:Male ratio: 47:53	Emotional item scores not substantially different between ethnic groups, but somatic item scores were. "Weak or sinking heart" "Aches and pains all over the body" and "Feeling of heat inside your body" were predictors of Asian ethnicity. These all relate to Urdu or Punjabi idioms.	B
Silveira & Ebrahim (1995)	Journal article	Quantitative	Bengali and Somali people aged over 60, London, UK	N=75<br>Ethnicity: Bangladeshi (100%)<br>First generation migrants: 100%<br>Age: range=60-80; mean=64<br>Female:Male ratio: 31:69	"Seventy-seven per cent of Bengalis complained of having pain in the neck, half of these 'a lot or unbearably', and the same proportion reported 'pounding in the heart' and/or breathlessness. Eighty per cent of those interviewed said they had been 'low in spirits and sat for ages doing absolutely nothing', 48% 'a lot or unbearably'. Approximately a third of Bengalis reported feelings of panic and being anxious. Forty four per cent had difficulty with sleeping and 63% 'worried about everything'. Most people expressed feelings of hopelessness regarding their future. Thirteen per cent of respondents reported suicidal thoughts, half of these ' a lot'" p.447	A
Upadhya (2015)	Thesis	Qualitative	Nepalese people aged 18-29 with personal experience of emotional distress or mental health difficulties, UK.	N=5<br>Ethnicity: Nepalese (100%)<br>First generation migrants: 100%<br>Age: range=19-29; mean=25<br>Female:Male ratio: 80:20	Headache; brain feeling "fuzzy"; persistent thoughts; "tension" (see Appendix 4); sleep disturbance; changes in appetite; cold sores; low mood; social withdrawal; guilt.	C

Author (Year)	Source type	Qualitative / Quantitative	Population of focus	South Asian Sample	Reported symptoms	Classification for review (Group A/B/C)
Yusuf (2019)	Thesis	Qualitative	People aged 18-65 of Pakistani, Indian, or Bangladeshi heritage, who have had experiences of emotional or psychological distress, Toronto, Canada.	N=15<br>Ethnicity: Pakistani (40%); Indian (40%); Bangladeshi (20%)	Fatigue; sleep disturbance; hopelessness; guilt; pain; helplessness; overwhelm; confusion; anger; social withdrawal; substance abuse; neglect of responsibilities.	A

Appendix 4: Uses of the term "tension"

Through the process of qualitative coding, we identified repeated use of the term "tension". This term was used by participants in studies of people of Pakistani, Indian, Bangladeshi, Punjabi, Nepalese, and non-specified "South Asian" heritage. [1–7]

We did not find that "tension" was presented a symptom of depression. However, common use of "tension" to refer to aspects mental distress means that culturally competent mental health care for the South Asian diaspora may be supported by understanding meanings invoked by this term. We therefore include here a short summary of our qualitative findings relating to meanings of "tension" from reviewed papers, combined with insights from the PAPER Study Patient and Public Involvement (PPI) Group.

	Notes	Example quotes
**Tension as ongoing stress or worry**	The PAPER Study PPI group indicate that "tension" is used in Pakistan to refer to ongoing substantial worry.<br><br>This perspective is supported by quotations from Pakistani participants in reviewed papers, including both Urdu and Punjabi speakers.[3,7] Some Pakistani participants described themselves as 'in tension' or 'having tension', invoking an ongoing/extended state, rather than a passing feeling.	*"These financial hardships created too much tension in our life."*[7] *(Punjabi Pakistani participant)*<br><br>*"He [my husband] is without money…That's why I was in severe tension."*[3] *(Pakistani participant)*<br><br>*"I already have tension"*[1] *(Pakistani participant)*<br><br>*"Gita explains that "tension" is a term commonly used by Nepalese people to vaguely describe the experience of stress. She describes how noticing*

	A UK Nepalese participant defined "tension" as a term to describe the experience of stress and internal struggle.[5]	*"tension" becomes a way of identifying and communicating the experience of an internal struggle."[5] (Author's paraphrasing of a Nepalese participant)*
**Tension as a cause of illness**	Nepalese participants reported that unresolved tension could be a cause of *paagal* (crazy),[5] while Nepalese, Pakistani and Indian participants identified tension as a cause of physical illness and somatic symptoms.[5,7]	*"The longer you keep experiencing tension and thinking about all the bad things that are happening, people start to become crazy" [5] (Nepalese participant)*<br><br>*"From this tension blood pressure will happen, and from blood pressure other illnesses will happen. Like these heart attacks and things, they are all because of tension"[5] (Nepalese participant)*<br><br>*"My nerves used to hurt due to tension" [7] (Indian participant)*<br><br>*"Due to staying in a continuous tension most of the time, my health started to decline, and I was diagnosed with ulcer and high blood pressure. [7] (Pakistani, participant)*

References

1. Malik R. *Depression kills more than a self: Concepts of mental distress amongst Pakistanis*. Doctoral, UCL (University College London), https://discovery.ucl.ac.uk/id/eprint/10098978/ (1997, accessed 18 November 2024).

2. Yusuf H. *Representations of psychological distress among Canadian Muslims of South Asian origin: A qualitative study using the self-regulatory model*. University of Toronto, https://www.academia.edu/68431581/Representations_of_Psychological_Distress_Among_Canadian_Muslims_of_South_Asian_Origin_A_Qualitative_Study_using_the_Self_regulatory_Model (2019).

3. Agarwal-Narale T. *Mental health of South Asian women: Dialogues with recent immigrants on post-migration, help-seeking and coping strategies*. McGill University, https://escholarship.mcgill.ca/concern/theses/m326m223p (2005).

4. Bottorff JL, Johnson JL, Venables LJ, et al. Voices of Immigrant South Asian Women: Expressions of Health Concerns. *Journal of Health Care for the Poor and Underserved* 2001; 12: 392–403.

5. Upadhya B. *How do young Nepalese people living in the UK make sense of mental health and problems of mental health: A qualitative exploration*. DClinPsy, University of Surrey, https://openresearch.surrey.ac.uk/esploro/outputs/99514910802346 (2015).

6. Mallinson S, Popay J. Describing depression: ethnicity and the use of somatic imagery in accounts of mental distress. *Sociology Health & Illness* 2007; 29: 857–871.

7. Akram S. *A qualitative study of the process of acculturation and coping for South Asian Muslim immigrants living in the Greater Toronto Area (GTA)*. University of Toronto, https://utoronto.scholaris.ca/server/api/core/bitstreams/6da433f1-66b4-4ca1-8339-f82aef00d5ef/content (2012).
